# Tales of the unexpected

**DOI:** 10.7554/eLife.87444

**Published:** 2023-04-04

**Authors:** Nicole Aponte Santiago, Stephanie Konecki, Lauren Porter, Harvinder Virk

**Affiliations:** 1 Department of Obstetrics, Gynecology and Reproductive Sciences, University of California, San Francisco San Francisco United States; 2 CG Life Chicago United States; 3 National Library of Medicine and National Heart, Lung, and Blood Institute, National Institutes of Health Bethesda United States; 4 University of Leicester Leicester United Kingdom

**Keywords:** sparks of change, research culture, careers

## Abstract

A single event can completely change the direction of a career in science; four researchers share their stories.

Most people working in research can expect to experience twists and turns in their career, from experiments that don’t work to bizarre comments from peer reviewers. There are times, however, when the path ahead seems to disappear altogether, leaving the researcher to wonder what they should do next. Sparks of Change hears from four scientists about times when they felt like their careers risked unravelling, prompting them to rethink their plans and end up somewhere unexpected.

## My typos led me to change fields

*Stephanie Konecki is a Scientific Content Specialist at CG Life, Chicago, United States*.

“That’s the fifth spelling error on this slide,” my (understandably) frustrated mentor mumbled. I gulped, humiliated, as I looked around the packed auditorium. This was my practice PhD thesis defense, and while the science was sound, the spelling was anything but.

For as long as I can remember, spelling and commas have been my Achilles’ heel. Until now, I could hide behind the red-squiggle safety net of spellcheckers. But the fancy new software I used to prepare the figures for my thesis defense did not come with my trusted ally. As my presentation came to an end, my mentor casually stated she thought I might be dyslexic.

My immediate thought was indignant: weren’t people diagnosed with dyslexia as children? Surely it was not possible to get to the end of a PhD without knowing you have the condition.

Despite these feelings, I went ahead and got tested. When the diagnosis came in, I felt more relieved than shocked. Things that had bothered me for decades instantly clicked. It finally made sense that I struggled to place myself on a map. There was a reason I couldn’t remember if I lived at 3415 or 1534 Sheridan Road. After years of poor math grades from swapped numbers, dropped negatives and misplaced decimals, I finally stopped thinking that I was broken, bad or lazy. I was just dyslexic.

With the diagnosis came tools and resources that set me free. It felt like putting on glasses for the first time: writing was an exciting storytelling adventure rather than a wrestling match with rogue commas. With my new tools, I gave myself permission to fall in love with writing.

The world can be a cruel and unforgiving place for people with dyslexia, making me hesitant to share this story. And yet, I wish I could go back in time and tell that girl in the auditorium that everything will be all right. Her work is much more than the red squiggle under a misspelled word.

I am now a full-time writer with a roster filled with happy clients, something I would have never thought possible pre-diagnosis. Being dyslexic does not stop your writing from being clever, creative or engaging. I hope this account encourages everyone to embrace their love for words because dyslexic or not, your story is worth telling.

## Seven minutes that *nearly* killed my dream

*Lauren Porter is a Stadtman Tenure-Track Investigator at the National Library of Medicine and the National Heart, Lung, and Blood Institute, National Institutes of Health, Bethesda, United States*.

Toward the end of my postdoc, I applied for an NIH ‘pathway to independence award’ that would help me to one day start my own lab: after years of lacking the confidence to become an independent investigator, I was finally ready to take the plunge. This was my last and only chance, as I was about to age out of the eligibility window required to apply for this type of award. I spent months painstakingly conceiving and writing the proposal, and many of my colleagues who reviewed it were optimistic it would be a success.

After all this work, only one step remained: for an administrative official to submit the proposal on my behalf. However, due to an issue on their end, my application cleared the NIH’s grant system seven minutes after the deadline. We immediately contacted the NIH to explain the issue, hoping they would excuse the short delay. A week later, the final decision reached my inbox: my proposal would not be considered.

I was devastated. Worse, with less than a year of funding left to support my current postdoc position, I was running out of time. My graduate mentor recommended that I look at the Howard Hughes Medical Institute’s Janelia Research Campus, which supports many scientists pursuing independent research. Heartened by his suggestion, I applied and was fortunate to be offered a research scientist position that enabled me to work without the time pressure of a fixed-term contract.

I approached this new opportunity with hope, along with a little uncertainty. My science focused on a very different research field, and I wasn’t sure how it would be received. Fortunately, I had an amazing mentor who supported the work I had proposed in my ill-fated grant application. As an added bonus, I got to work with scientists from a range of disciplines who broadened my research horizons. Although I couldn’t foresee it at the time, an administrative blunder that could have ruined my career instead put me on the path to become a better scientist, ultimately allowing me to get my current position and realize my dream of becoming an independent investigator.

## My model organism and me were not meant to be

*Nicole Aponte Santiago is in the Department of Obstetrics, Gynecology and Reproductive Sciences, University of California, San Francisco, San Francisco, United States*.

My PhD focused on using fruit flies to study how two classes of motoneurons behave. About a year in, however, I started to feel sick after spending time in the room where the flies were stored. Initially, my sniffles and watery eyes would fade by the end of the day, but I steadily started to feel more tired and was struggling to concentrate. After weeks of these symptoms, I came to a strange realization: I might have developed an allergy to my model organism!

If this were true, what could it mean for my research? Would I have to abandon my graduate project just as it was picking up steam? Worried, I turned to academic Twitter to see if anyone else had experienced this before. I was relieved to find a community of researchers studying fruit fly allergies, including professors doing fantastic work with flies who had found ways to control their symptoms.

Before I could implement these tried and tested strategies, I needed to get an allergy test to confirm fruit flies were causing my symptoms. I homogenized a sample of the lab’s flies to take with me to the doctor, along with the only paper I could find on fly allergies (which my physician also showed up with!). As expected, my arm turned red right where the fly allergen had been applied, confirming my suspicions.

Thankfully, my lab and institute responded quickly to provide me with the right equipment and accommodations to minimize my symptoms. I also changed the way I planned experiments to spend as little time as possible in the fly room, which boosted my productivity. This meant that by the time the first lockdown hit in 2020, I was fortunate to already have the data I needed to start writing my thesis.

In the end, my unlucky fly allergy had a productive outcome. Still, for my postdoc, I thought it was best not to risk it again, and I now study neurons in zebrafish.

## The bad antibodies that shook my faith in science

*Harvinder Virk is a NIHR Academic Clinical Lecturer in Respiratory Medicine at the University of Leicester, Leicester, United Kingdom*.

Nearly seven years ago**,** I received funding for a PhD investigating if an ion channel called TRPA1 was important in asthma. The project relied on an antibody which is meant to stain the channel in tissue biopsies – the same antibody I had used to generate some of the preliminary data in my application. At the time, a reviewer suggested I should check the antibody was staining the right protein. I was surprised by this comment. After all, the reagent was manufactured by a reputable company and many influential papers had used it.

The validation experiments took several months to complete, and my heart sank as the results started to come in. The antibody was not even weakly detecting TRPA1; instead, it was staining an unrelated protein. By then my work on the patient biopsies was already well under way and showing exciting findings – none of which were valid.

Sharing this discovery with my supervisors and colleagues was difficult, despite their constant support. I felt guilty for all the time and resources I had wasted, and I still do now. We didn’t want to believe what I had found, but it soon became undeniable. None of the five antibodies for TRPA1 available on the market effectively stained the channel in our biopsies.

At the time, I felt I had discovered a scandal. However, it soon became clear to me that this was not limited to TRPA1, and many researchers working on other proteins had faced the same issue with their antibodies. I started to lose faith in the research process; I briefly contemplated leaving academia. Things changed when I discovered the work of people, such as Carl Laflamme and the YCharOS team, who are raising awareness of the problem. I could see that slow progress was being made, and I wanted to be part of it. This gave me the confidence to publish my own validation results, which became the first paper of my PhD.

In the end, I was able to change the course of my PhD and study the role of TRPA1 in more reliable ways, allowing me to secure my current position. I have not left the topic of antibody validation behind, however, and my team was recently awarded a small grant to understand the research culture factors that contribute to this issue. Seeing mine as well as other’s validation work being used to explain why certain important results in the literature cannot be replicated has helped restore my faith in the self-correcting nature of science.

## Share your experiences

This article is a Sparks of Change column, where people around the world share moments that illustrate how research culture is or should be changing. Have an interesting story to tell? See what we’re looking for and the best ways to get in touch here.

